# Image-Based Hot Pepper Disease and Pest Diagnosis Using Transfer Learning and Fine-Tuning

**DOI:** 10.3389/fpls.2021.724487

**Published:** 2021-12-16

**Authors:** Yeong Hyeon Gu, Helin Yin, Dong Jin, Jong-Han Park, Seong Joon Yoo

**Affiliations:** ^1^Department of Computer Science and Engineering, Sejong University, Seoul, South Korea; ^2^Horticultural and Herbal Crop Environment Division, National Institute of Horticultural and Herbal Science, Rural Development Administration, Wanju, South Korea

**Keywords:** deep feature, distance metric, fine-tuning, hot pepper, *k*-nearest neighbors, transfer learning

## Abstract

Past studies of plant disease and pest recognition used classification methods that presented a singular recognition result to the user. Unfortunately, incorrect recognition results may be output, which may lead to further crop damage. To address this issue, there is a need for a system that suggest several candidate results and allow the user to make the final decision. In this study, we propose a method for diagnosing plant diseases and identifying pests using deep features based on transfer learning. To extract deep features, we employ pre-trained VGG and ResNet 50 architectures based on the ImageNet dataset, and output disease and pest images similar to a query image *via* a *k*-nearest-neighbor algorithm. In this study, we use a total of 23,868 images of 19 types of hot-pepper diseases and pests, for which, the proposed model achieves accuracies of 96.02 and 99.61%, respectively. We also measure the effects of fine-tuning and distance metrics. The results show that the use of fine-tuning-based deep features increases accuracy by approximately 0.7–7.38%, and the Bray–Curtis distance achieves an accuracy of approximately 0.65–1.51% higher than the Euclidean distance.

## Introduction

Hot peppers comprise one of the world’s most popular crops. In 2018, the Food and Agricultural Organization reported that production of hot-pepper (item: “chiles and pepper, green”) had steadily increased to approximately 36.8-million tons, up more than 14.4% compared with 2014 [Food and Agriculture Organization [FAO], 2018]. Hot-pepper production is greatly affected by climate change (Aji et al., 2020), and owing to increased importing and exporting, the influx of foreign diseases and pests are prominent threats.

Past studies of plant disease and pest recognition used classification methods that presented a singular recognition result to the user. Unfortunately, incorrect recognition results may be output, which may lead to further crop damage. Therefore, there is a need for a system that can offer multiple candidate results so that the user can intervene and weigh options. Google (2020) presents several candidate results to an image query and allows the final selection to be made by the user. The content-based image retrieval (CBIR) technique can also be used for this purpose. CBIR extracts features by applying a specific content (e.g., color and edge) descriptor to an image, and it outputs the most similar images to a query image using similarity comparison between features. However, owing to limitations of the feature-extraction descriptor, the recognition accuracy of diseases and pests is low at ∼75–83% (Yin et al., 2016, 2020; Piao et al., 2017). Thus, it is necessary to improve recognition performance using a deep-learning algorithm.

In cases where there are insufficient data, or models are not well-trained, transfer learning can be used (Kaya et al., 2019; Deng et al., 2020; Zhuang et al., 2021). Many studies on machine vision have employed transfer learning. It has been widely applied to solve problems related to image recognition using convolutional neural network (CNN) models. Typically, copious data, time dimensions, and computing resources are required to train models with deep layers. An example is the visual geometry group (VGG) (Simonyan and Zisserman, 2014) and ResNet (He et al., 2016) models. These architectures have already shown excellent image verification performance with various large public datasets.

Transfer learning is a machine-learning methodology that focuses on knowledge transfer between domains. It can be quickly applied to tasks using pre-trained knowledge (Tsiakmaki et al., 2020; Zhuang et al., 2021). Thus, the number of cases using transfer learning to recognize diseases and pests is increasing. Too et al. (2019) analyzed the performance of plant-disease identification using fine-tuned VGG, Inception, ResNet, and DenseNet models. Their research findings showed that DenseNet achieved the best performance. Sagar and Dheeba (2020) conducted research on the recognition of plant diseases using the Plant Village dataset (Hughes and Salathe, 2015). Their results showed that ResNet50 with a skip-connection structure achieved a recognition accuracy of 98.2%. Rangarajan and Raja (2020) conducted research on the classification of 10 diseases related to four crops: eggplant, hyacinth beans, lime, and ladies’ finger (okra). They employed six pre-trained CNN architectures, including AlexNet and VGG16. The results showed that GoogLeNet achieved the highest verification accuracy of 97.3%. Dawei et al. (2019) proposed a pest-diagnostic system using transfer learning. For this, they developed a deep-learning model capable of classifying 10 pest images and compared its performance with human experts and traditional neural-network-model training methods. The results of the proposed method showed performance results similar to those of human experts and a classification accuracy of 93.84%. Pattnaik et al. (2020) presented a pre-trained CNN-based transfer-learning framework for tomato-pest recognition. Their research used 859 images collected online, classified into 10 classes. They performed transfer learning using 15 pre-trained models, and the experimental results showed that the DenseNet169 model achieved the best performance with a classification accuracy of 88.83%. Leonardo et al. (2019) identified fruit flies using nine machine-learning techniques and deep features extracted by five models, including VGG and inception, by applying transfer learning. The method of applying deep features extracted using the VGG16 model to a support vector machine (SVM) achieved the best accuracy of 95.68%. Aravind et al. (2019) applied transfer learning to disease-image classification for grapes. They pre-trained AlexNet on the PlantVillage dataset and trained a multiclass SVM (MSVM) model using the deep features extracted from each AlexNet layer as image features. The results showed that the best performance was achieved when the features extracted from the third rectified linear unit layer of the AlexNet model were applied to the MSVM model. In that research, fewer than 100 images per class were used when training the deep-learning model, but high recognition accuracy was achieved through transfer learning.

Yin et al. (2020) proposed a disease and pest recognition method using deep features based on transfer learning, achieving recognition accuracies of 85.6 and 93.62% for the top-10 results of hot-pepper diseases and pests, respectively. However, in their study, they extracted features using pre-existing weights without a tuning process for the pre-trained model. In this study, we propose an improved method for diagnosing diseases and pests using fine-tuning based on those previous studies. Furthermore, we demonstrate the excellence of the proposed model by measuring the following effects through various experiments:

• Performance comparison when fine-tuning the last dense layer and the conv+dense layer in the classification model;
• Effect of fine-tuning on deep features;
• Effect of the distance metric on the proposed model;
• Performance comparison between the conventional classification model and the proposed disease and pest diagnosis model.

## Materials and Methods

### Dataset Description

**FIGURE 1:**
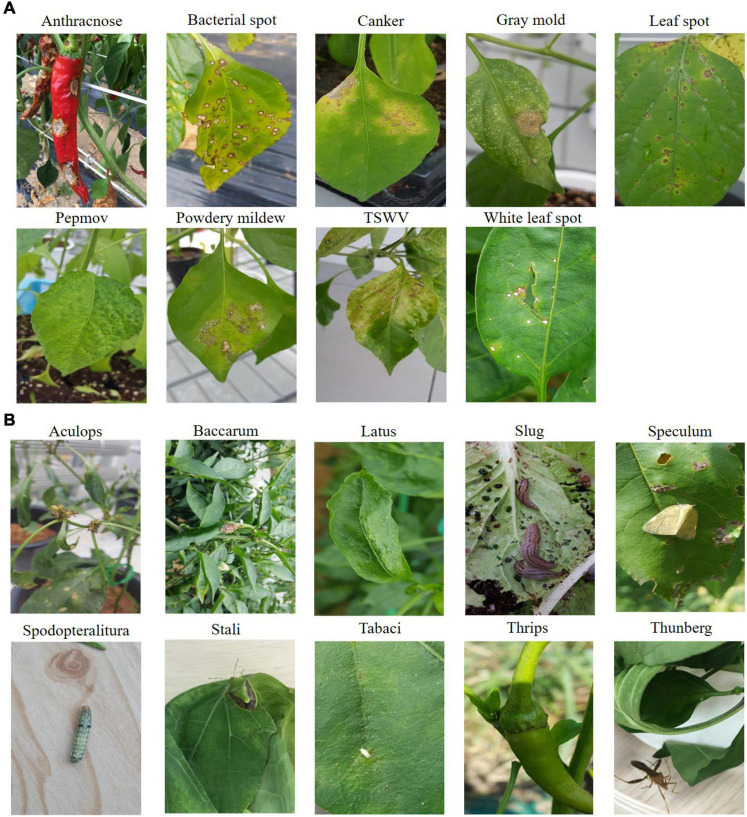
Examples of disease/pest classes.

In this study, we used hot-pepper disease and pest images provided by the National Institute of Horticultural and Herbal Science. Figure 1A shows sample images of the diseases, and Figure 1B displays those of pests. In the experiments, we used 23,868 cropped disease and pest images (disease: 15,435; pest: 8,433) for 19 types (disease: 9; pest: 10) (Tables 1, 2).

**TABLE 1:** Summary of hot pepper disease dataset.

Diseases	Original images	Cropped images	Training samples	Testing samples
Anthracnose	283	1,152	1,037	115
Bacterial spot	161	2,015	1,814	201
Canker	144	660	594	66
Gray mold	171	2,304	2,074	230
Leaf spot	291	1,526	1,374	152
Pepmov	42	1,281	1,153	128
Powdery mildew	278	3,146	2,832	314
TSWV	106	1,037	934	103
White leaf spot	221	2,314	2,083	231
Total	1,697	15,435	13,895	1,540

**TABLE 2:** Summary of hot pepper pest dataset.

Pests	Original images	Cropped images	Training samples	Testing samples
Aculops	139	1,140	1,026	114
Baccarum	90	684	616	68
Latus	46	720	648	72
Slug	138	1,212	1,091	121
Speculum	623	1,152	1,037	115
Spodopteralitura	167	633	570	63
Stali	58	696	627	69
Tabaci	78	540	486	54
Thrips	60	1,008	908	100
Thunberg	51	648	584	64
Total	1,450	8,433	7,593	840

**FIGURE 2:**
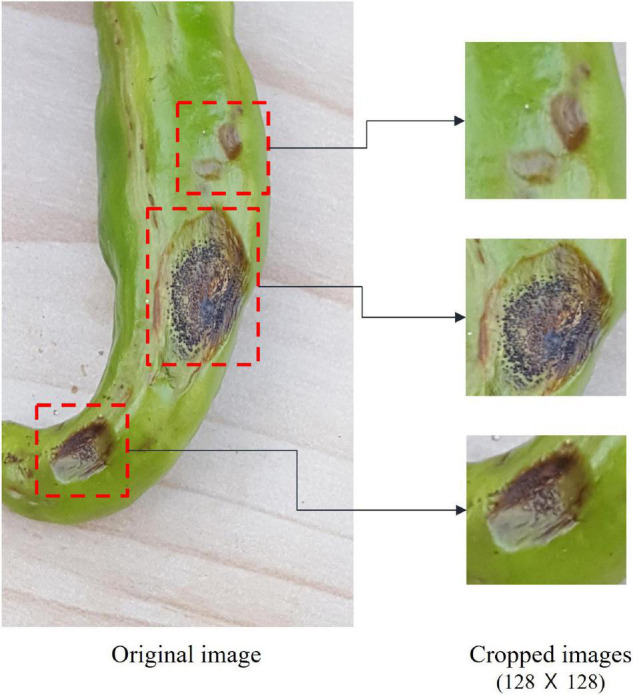
The process of image cropping.

In this study, instead of using the original disease and pest images, we used a cropped set containing the diseased areas. Image cropping reduces image recognition time and improves accuracy (Suh et al., 2003; Chen et al., 2016). The image cropping performed in this study was manually performed by agricultural experts to select the diseased areas as accurately as possible. As seen in Figure 2, we created at least one cropped image of 128 × 128 pixels from each original.

### Pre-trained Models

In this study, different pre-trained models have been used as a transfer learning such as VGG16, VGG19 (Simonyan and Zisserman, 2014) and ResNet50 (He et al., 2016). The reason for using these three pre-trained models is that they are the top three models that showed the highest performance in the previous study (Yin et al., 2020). A pre-trained model is a network that was trained on a large dataset. Such a pre-trained model, for example ImageNet, can overcome insufficient training data, and it has high flexibility, because a model suitable for a particular task can be created by fine tuning it (Tan et al., 2018; Kaya et al., 2019). The pre-trained ImageNet model classifies 1,000 classes. Therefore, we had to modify it to our problem. In this study, pre-trained VGG16, VGG19 and ResNet50 models were used for transfer learning.

### Transfer Learning of Deep Convolutional Neural Network

Transfer learning is a machine-learning method that focuses on the application of knowledge acquired from solving existing problems to solve new problems. It is extensively used for computer vision and natural language processing applications. It can achieve high accuracy in a relatively short time (Rawat and Wang, 2017). In particular, transfer learning can efficiently solve problems when only a small number of data is available, or huge computing and time resources are needed (Tan et al., 2018; Noor et al., 2020). ImageNet is the most extensively used for pre-training. It consists of 21,841 classes of approximately 14-million images. Of these, a sub-dataset of 1,000 classes is commonly used for benchmarking (Russakovsky et al., 2015). CNN architectures trained using ImageNet include VGG, ResNet, Inception (Szegedy et al., 2015), Xception (Chollet, 2017), and Densenet (Huang et al., 2017). Of these, we employed VGG and Resnet models.

#### Visual Geometry Group Model

**FIGURE 3:**
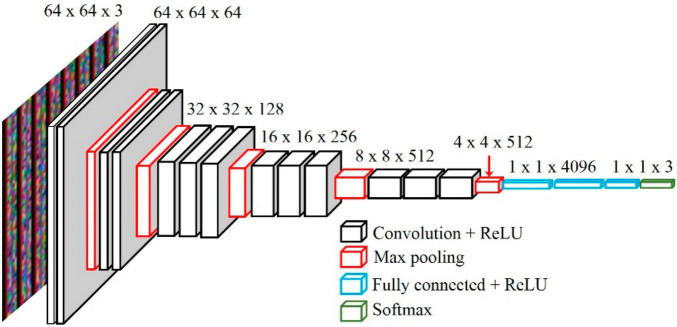
The architecture of the pre-trained VGG16 (Ullah et al., 2020).

The VGG network is a CNN model devised by Simonyan and Zisserman (2014) for the 2014 ImageNet Large Scale Visual Recognition Challenge (ILSVRC). Although the VGG model is a bit heavy, its structure is simple (Canziani et al., 2016). The model supports deep layers made by stacking convolutional and pooling layers in a certain pattern. A 3 × 3 kernel is used in the convolutional layer, and the height and width of the input and output feature maps are set to the same by using a stride value of one. In the pooling layer, the height and width of the feature map are reduced by half through a 2 × 2 stride-two max-pooling operation. The architecture is shown in Figure 3 as VGG16 or VGG19, depending on the depth of the model.

#### ResNet Model

**FIGURE 4:**
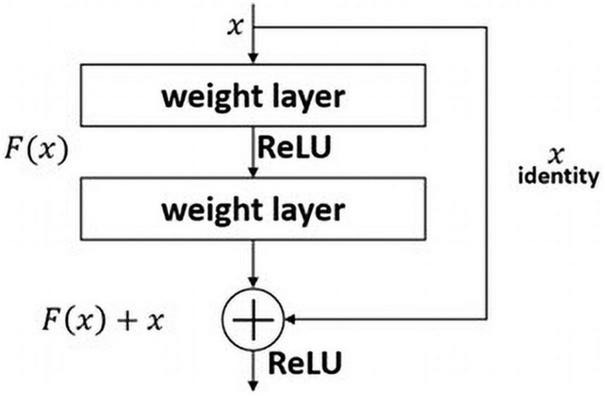
Skip connection.

The ResNet model won the 2015 ILSVRC. The most significant difference between other model is that the number of layers in the ResNet architecture is sharply deeper than that of existing models. VGG has 16 or 19 layers, GoogLeNet has 22, and ResNet has 152. The deeper the layer of the deep-learning model is difficult to train because the larger the number of weights. And gradient vanishing issue can also occur (Bengio et al., 1994; Glorot and Bengio, 2010; Rawat and Wang, 2017). ResNet addresses these issues by using a residual block. As seen in Figure 4, the residual block makes it possible to effectively transfer the gradient between layers using a skip connection. This is similar to the philosophy of long short-term memory in recurrent neural networks, used to better transfer the gradient of the previous step through a forget gate (Staudemeyer and Morris, 2019). In this study, we employ a pre-trained ResNet50 model using the ImageNet dataset instead of the 152-layer ResNet.

#### Feature Extraction and Fine-Tuning

Strategies using transfer learning are mainly divided into feature extraction and fine-tuning (Kandel and Castelli, 2020; Luján-García et al., 2020). Feature extraction involves extracting features from new samples using a representation of the pre-trained network. Using the extracted features, a classification model can be obtained that fits the problem by training a new classifier from scratch. The CNN model comprises consecutive convolution and pooling layers and performs classification through fully connected layers. Feature extraction regards the output value of a specific layer of the pre-trained model as a feature, and the feature extracted from the deep-learning model is called a deep feature.

Fine-tuning refers to the method of transforming an architecture for a new purpose based on a pre-trained model and updating training from the pre-trained weights. This method adjusts some of the representations of the reuse model to be closer to the given problem. The process of fine-tuning is as follows:

(1) Add architecture (layer or network) to the pre-trained base network;
(2) Freeze the base network;
(3) Train the newly added layer or network;
(4) Unfreeze some layers in the base network;
(5) Train the unfrozen and newly added layers with new data.

In this study, we adopt a more concise method. See section “Layer Freezing and Fine-Tuning.”

### *k*-Nearest-Neighbor Algorithm

**FIGURE 5:**
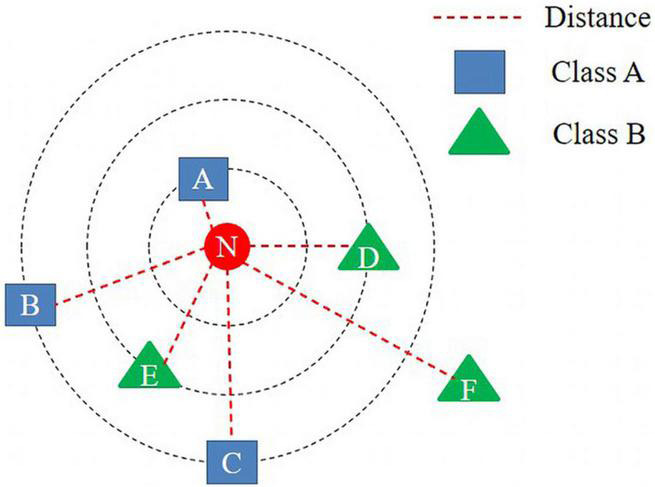
Illustration of how *k*-nearest neighbors’ algorithm works.

The *k*-nearest-neighbor (kNN) algorithm is a supervised learning method that classifies unlabeled observations based on the most-similar labeled examples in the attribute space (Zhang, 2016). During classification, this algorithm refers to the information of *k* instances around a given point and makes the final decision *via* majority voting. For example, as shown in Figure 5, there are six instances of two classes (A and B) in the vector space. Here, we intend to classify the class of N when a given point is input into the vector space. The kNN algorithm calculates the distance between the input data and all other data without creating a separate model. Next, the class of the input data is determined by referring to the information of *k* instances around them. For instance, when *k* is set to one in Figure 3, the input data are classified as Class A, because the distance between the input data, N, and point A is the shortest. When *k* is set to three, it is ultimately classified as Class B, because it refers to points A, D, and E. As such, classification performance varies, depending on the value of *k*. Thus, choosing the right value of *k* is crucial (Koklu and Ozkan, 2020).

Apart from the value of *k*, a critical distance function calculates the similarity between vectors. This function has the advantage of effectively handling high-dimensional data and reducing computation time. Thus, the use of a suitable distance function can improve model performance. The most commonly used distance function is the Euclidean distance, shown in Equation 1.


(1) $$D_{L\,2\,\left(x,y\right)}=\sqrt{\sum_{i=1}^{n}{\left(x_{i}-y_{i}\right)}^{2}}.$$

## Deep-Learning Methodologies

### Data Preprocessing and Augmentation

The hot-pepper disease and pest images used in this study were cropped images of 128 × 128 pixels. To use the pre-trained model on the ImageNet dataset, we performed pre-processing on the disease and pest images the same as we did for training the ImageNet dataset. First, we resized the disease and pest images to 224 × 224 pixels. As in the study by Simonyan and Zisserman (2014), we normalized the images by calculating the average value of each channel of the dataset. Then, we subtracted the average value calculated for each input image. The average value was a 1D array containing the average values of RGB pixels of the entire ImageNet image: 103.939, 116.779, and 123.68, respectively.

### Layer Freezing and Fine-Tuning

Layer freezing prevents the layer weight from being modified. This technique is often used with transfer learning and fine-tuning, where the base model or lower layer trained on another dataset is frozen. Training can then be accelerated by using an appropriate freezing technique (Brock et al., 2017). In this study, we froze most layers of the VGG and ResNet models and performed fine-tuning on the last convolution layer.

To fine-tune the pre-trained VGG16, VGG19 and Resnet50 models, we removed the existing fully connected layer. Then added a dense layer (VGG model: 512 nodes, Resnet50: 2,048 nodes) and a new softmax layer that fit our data classes (i.e., diseases: 9; pests: 10). It was set equal to the number of values from the last convolutional layer. Thus, the newly added dense layer’s node was 512, and 2,048 for Resnet50. We performed fine-tuning in two different ways: (i) only fine-tune the newly added dense and softmax layer, (ii) fine-tune the last convolution layer, dense and softmax layer. And the rest of the layers were frozen.

The batch size was set to 256, and a categorical cross-entropy loss function was used. Stochastic gradient descent was used as an optimizer, and a learning rate of 0.001 was used in this study. The epoch was set to 500, and early stopping was added to avoid over-fitting problems. The early stopping was set to terminate the training when the validation accuracy did no longer improve over the next 20 times.

In this study, fine-tuned models were used as a feature extractor. Remove the softmax layer from fine-tuned model and use the value from the newly added dense layer as deep feature. Because the architecture of the VGG and the Resnet model are different, the dimension of deep feature is also different. Deep features extracted from VGG16 and VGG19 models have a dimension of 512, and 2,048 dimensions from Resnet50.

### Proposed Method

**FIGURE 6:**
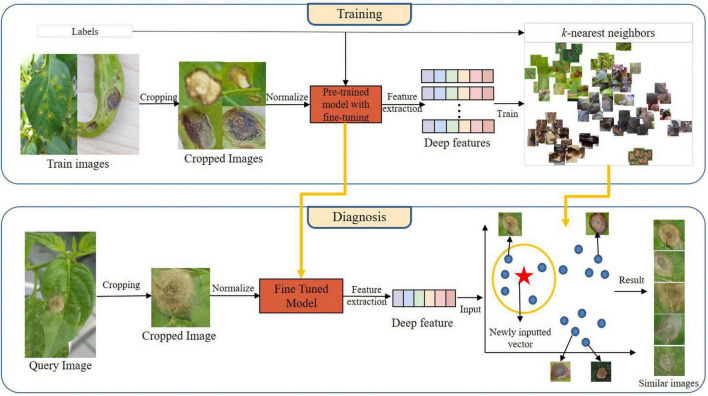
The architecture of the proposed diagnostic model.

The proposed architecture comprising training and diagnosis processes is shown in Figure 6. The training process mainly consisted of image cropping, fine-tuning the pre-trained model, deep feature extraction, and kNN-algorithm training. In this study, we extracted deep features of cropped images using the fine-tuned pre-trained model, and we represented the extracted deep features in the vector space by using kNN algorithm.

In the diagnosis process, we extracted deep features by given cropped images into the fine-tuned model generated during the training process. The extracted deep features were input into the trained kNN model, which output the *k* vectors most similar to itself in the vector space. Here, each vector refers to a cropped image. Five similar images were output for each query image by setting the value of *k* to five.

We used the Bray–Curtis distance (Bray and Curtis, 1957) as the crucial kNN distance metric to improve the diagnostic accuracy of the proposed model. The Bray–Curtis distance provides a normalization method commonly used in the fields of ecology and environmental science. The distance between Vectors A and B can be calculated using Equation 2, referring to a vector of length N. The Bray-Curtis distance has a value ranging from zero to one. As it approaches zero, it indicates that they are closer together.


(2) $$\mathrm{Bray}\,\mathrm{Curtis}\,\mathrm{distance}\,\left(A,B\right)=\frac{\sum_{i=1}^{N}\left|A_{i}-B_{i}\right|}{\sum_{i=1}^{N}A_{i}+\sum_{i=1}^{N}B_{i}}.$$

## Experimental Results

### Tools and Setup

Experimental work was performed using Python v.3.6 on a Windows desktop with two Nvidia GeForce RTX 2080 Ti graphical processing units. We divided the dataset into a training set and a test set to fine-tune the pre-trained models. The training and test sets were randomly chosen from each category, with 90% and 10% ratios, respectively.

### Measurement Criteria

In this study, we applied two indices (i.e., precision and accuracy) to measure the performance of classification method and proposed method, as shown in Equations 3, 4. Equation 3 measured the performance of the classification model, and Equation 4 measured the performance of the proposed diagnostic model:


(3) $$p\,r\,e\,c\,i\,s\,i\,o\,n=\frac{1}{N}\,\sum_{i=1}^{N}\frac{T\,r\,u\,e\,p\,o\,s\,i\,t\,i\,v\,e}{T\,r\,u\,e\,p\,o\,s\,i\,t\,i\,v\,e+F\,a\,l\,s\,e\,p\,o\,s\,i\,t\,i\,v\,e}$$

(4) $$a\,c\,c\,u\,r\,a\,c\,y=\frac{1}{N}\,\sum_{i=1}^{N}\frac{\left|\left\{r\,e\,l\,e\,v\,a\,n\,t\,i\,m\,a\,g\,e\,s\right\}\,⋂\left\{r\,e\,t\,r\,i\,v\,e\,d\,i\,m\,a\,g\,e\,s\right\}\right|}{\left|\left\{r\,e\,t\,r\,i\,e\,v\,e\,d\,i\,m\,a\,g\,e\,s\right\}\right|}$$

Equation 4 provides an index for measuring the performance of the proposed diagnostic method. Here, among the output results, the relevant images were determined as those having the same class as the query. Five retrieved images became the output images. *N* refers to the number of images included in each disease and pest class in the test image set, and *i* refers to the index number of each query image. Thus, the accuracy index represents the proportion of images having the same class as a query image among similar images.

### Result and Discussion

We investigated the following effects through experimentation:

• Performance comparison when fine-tuning the last dense layer and the conv+dense layer in the classification model;
• Effect of fine-tuning on deep features;
• Effect of the distance metric on the proposed model;
• Performance comparison between the conventional classification model and the proposed disease and pest diagnosis model.

#### Effect of Fine-Tuning According to a Specific Layer During Classification

**FIGURE 7:**
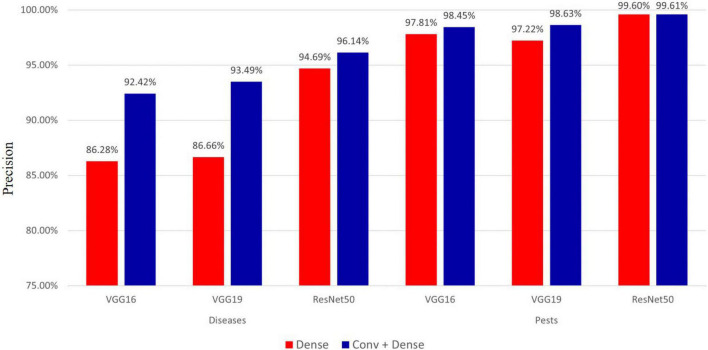
Precision comparison when fine-tuning dense layer and conv+dense layer.

Fine-tuning plays a crucial role in improving model performance. In this study, we compared the performance of the pre-trained VGG16, VGG19, and ResNet50 models when fine-tuning the last dense layer and the conv+dense layer, respectively, and the results are shown in Figure 7.

As seen in Figure 7, for hot-pepper diseases, the precision of fine-tuning the conv+dense layer was approximately 1.45–6.83% higher than that of the model in which only the dense layer was finely tuned. For hot-pepper pests, the precision of fine-tuning the conv+dense layer was approximately 0.01–1.41% higher than that of the model in which only the dense layer was finely tuned. Of the three pre-trained models, the ResNet50 model achieved the highest precisions of 96.14 and 99.61% for diseases and pests, respectively.

#### Effect of Fine-Tuning on Deep Feature

**FIGURE 8:**
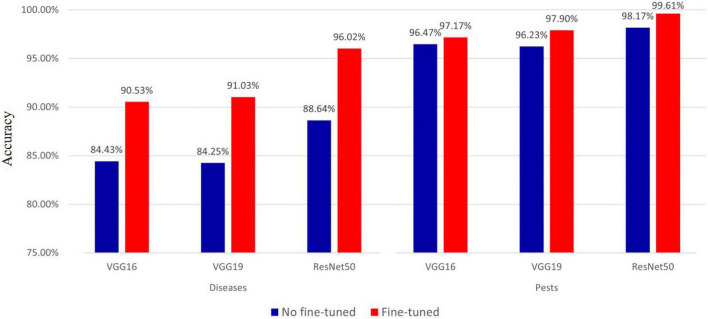
Accuracy comparison of fine-tuned and non fine-tuned models in hot pepper diseases and pests.

In the proposed diagnostic model, we used the deep features extracted from the pre-trained models. Therefore, we measured the effect of fine-tuning through the use of the deep features extracted from the finely tuned VGG16, VGG19, and ResNet50 models. The results of performance comparisons are shown in Figure 8. The results show that the accuracy of using the deep features extracted from the finely tuned model for hot-pepper diseases was approximately 6.1–7.38% higher than that of using the deep feature without fine-tuning. These results were also true for hot-pepper diseases, showing a higher accuracy of 0.7–1.67%. Furthermore, of the three pre-trained models, the ResNet50 model showed the highest performance for diseases and pests with accuracies of 96.02 and 99.61%, respectively.

#### Effect of Distance Metric on the Proposed Method

**FIGURE 9:**
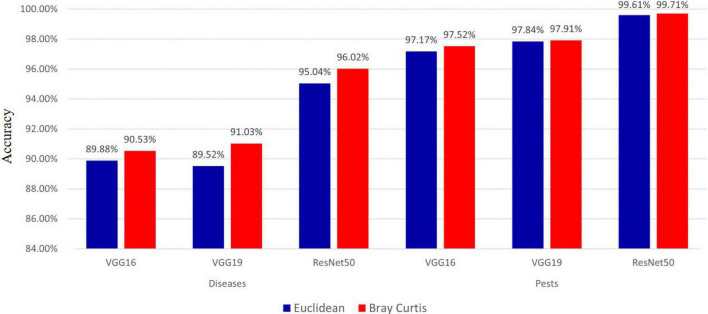
Accuracy comparison of distance metric.

Because the proposed diagnostic model used the kNN algorithm, the role of the distance metric that calculated the distance between vectors in the vector space was crucial. In this experiment, to measure the effect of the distance metric on the proposed method, we measured performance using two metrics (i.e., Euclidean and Bray–Curtis distances), and the results are shown in Figure 9. The results show that the accuracy of the Bray-Curtis distance was approximately 0.65–1.51% higher than that of the Euclidean distance for hot-pepper diseases. For hot-pepper pests, the accuracy of the Bray-Curtis distance was approximately 0.07–0.35% higher. These results demonstrated excellent Bray–Curtis distance.

#### Discussion

To reduce the result of incorrect recognition of the classification methods used in most previous studies, a method was needed that presents several candidate results of high probability to the user, allowing them to make the final decision. We proposed a disease and pest diagnosis model using a transfer learning and fine-tuning technique.

In the experiment described in section “Effect of Fine-Tuning According to a Specific Layer During Classification,” we compared the performance when fine-tuning specific layers (i.e., dense layer and conv+dense layer). As seen in Figure 7, we achieved the highest performance when fine-tuning the conv+dense layer. Performance was improved by approximately 0.01–1.41% for pests, whereas it was improved by approximately 1.45–6.83% for diseases. Despite using the same fine-tuning method, the reason for this difference in performance can be attributed to the dataset. Most pest images contain pests, and they have more distinct features than do disease images. On the other hand, disease images often have similar symptoms, despite different disease classes. In the convolution layer, image features were extracted through the convolutional and pooling layers. Therefore, it showed a greater effect on images having similar symptoms by fine-tuning the convolution layer.

We measured the effect of fine-tuning on the deep features used in the proposed model, as shown in Figure 8. The deep features to which fine-tuning was applied improved accuracy by approximately 0.7–7.38%, compared with fine-tuning not being used. This demonstrates the importance of fine-tuning.

Table 3 shows the results of comparing the performance of the proposed model in this study with the classification model using the fine-tuning method. The results show that the accuracy of the classification method was 97.88%, which was 0.01% higher than that of the proposed model. Although the performance of the classification as was higher, an incorrect result may be output with a probability of approximately 2.12%, because this is a single result. However, because the accuracy of the proposed model was the measurement of the weight of images having the same class as the query image among a total of five candidate groups, the error can be reduced *via* the final decision of the user. For example, because the average accuracy of the proposed model was 97.87% and assuming that 100 similar images were output, approximately 98 correct answers and two incorrect ones were presented to the user. With the proposed method, there is, therefore, a higher probability of reducing incorrect recognition results is provided, owing to expert human intervention.

**TABLE 3:** Performance comparison of single recognition method and proposed method.

	Single recognition method	Proposed method
Diseases	96.14%	96.02%
Pests	99.61%	99.71%
Average	97.88%	97.87%

## Conclusion

In this study, we proposed an improved method for diagnosing hot-pepper diseases and pests using a fine-tuning-based transfer learning method. To extract deep features, we employed pre-trained VGG16, VGG19, and ResNet50 models based on the ImageNet dataset and output disease and pest images most similar to the query image using the kNN algorithm. We used image data of 19 types of hot-pepper diseases and pests, and the experimental results showed that accuracies of 96.02 and 99.61% were achieved for diseases and pests, respectively. We also measured the effects of fine-tuning and distance metrics. The measurement results showed that fine-tuning improved the accuracy by approximately 0.7–7.38%, and the Bray-Curtis distance achieved a higher accuracy of approximately 0.65–1.51% than that of the Euclidean distance. Furthermore, when comparing the performance between the proposed model and the classification, they showed an accuracy performance of 97.87 and 97.88%, respectively. In summary, an expert user is expected to derive more accurate pest recognition results from the proposed model, which requires manual image cropping around the disease area. In the future, we will automatic the image cropping and measure its effectiveness by applying the proposed model to other crops.

## Data Availability Statement

The raw data supporting the conclusions of this article will be made available by the authors, without undue reservation.

## Author Contributions

HY, DJ, and YG: conceptualization. YG: methodology, writing – review and editing. HY: methodology, investigation, and writing – original draft. DJ: methodology, validation, and writing – review and editing. YG and J-HP: resources and supervision. SY: project administration. All authors contributed to the article and approved the submitted version.

## Conflict of Interest

The authors declare that the research was conducted in the absence of any commercial or financial relationships that could be construed as a potential conflict of interest.

## Publisher’s Note

All claims expressed in this article are solely those of the authors and do not necessarily represent those of their affiliated organizations, or those of the publisher, the editors and the reviewers. Any product that may be evaluated in this article, or claim that may be made by its manufacturer, is not guaranteed or endorsed by the publisher.
